# Compassion fatigue and compassion satisfaction among Romanian emergency medicine personnel

**DOI:** 10.3389/fmed.2023.1189294

**Published:** 2023-07-24

**Authors:** Anca Hăisan, Simona Hogaș, Cornelia Măirean, Mirabela-Olivia Punei, Simona Ruxandra Volovăț, Mihai Hogaș, Cristina Kantor, Diana Cimpoeșu

**Affiliations:** ^1^Faculty of Medicine, University of Medicine and Pharmacy “Grigore T. Popa”, Iași, Romania; ^2^Faculty of Psychology and Educational Sciences, Alexandru Ioan Cuza University, Iași, Romania

**Keywords:** secondary traumatic stress, burnout, compassion satisfaction, threats, worries

## Abstract

**Background:**

Contemporary scientific literature has emphasized two specific aspects of healthcare professionals: compassion satisfaction and compassion fatigue. In the context of the COVID-19 pandemic, which has placed significant strain on health systems and healthcare workers, the Russian-Ukrainian crisis appears to have a magnifying effect, particularly on mental health.

**Methods:**

The aim of the present study was to investigate the relationship between threat perception, daily worries, and professional quality of life in a sample of Emergency Medicine Personnel during two major events mentioned above. The sample included 372 participants (56.7% nurses and 43.3% physicians) from emergency units in five county hospitals in the Eastern region of Romania.

**Results:**

The study revealed that threats related to the pandemic were positively linked to secondary traumatic stress, and daily worries were positively linked to both secondary traumatic stress and burnout. Threats generated by the war did not manifest a direct relation with any of the indicators of professional quality of life, but daily worries generated by war positively predicted both secondary traumatic stress and burnout.

**Conclusion:**

Both the pandemic, which involved cumulative exposure, and the war, which involved a lower and more distant level of exposure, had the potential to generate worries and predict a low quality of life. However, our results did not reveal any association between threats, worries, and compassion satisfaction. As a result, this positive indicator of quality of life remained stable despite the presence of threats and worries.

## 1. Introduction

In emergency medicine, the constant high-stress environment and repeated exposure to the suffering of others make individuals more vulnerable to compassion fatigue, which has detrimental consequences for the individual, the patient, the workplace, and the healthcare system (1, 2). Compassion fatigue and compassion satisfaction represent two important dimensions of professional quality of life (3–5). Compassion fatigue is defined as the weakening of the psychological defense mechanisms employed by workers to respond to and deal with severe work-related stress factors (6, 7). It arises from the cumulative empathic engagement with others’ trauma, involving both cognitive and affective components (8, 9). This includes the risk of secondary traumatic stress (STS) and burnout. Individuals who suffer from secondary traumatic stress frequently find themselves reliving a traumatic event and display symptoms such as increased arousal, avoidance behavior, and unsettling patient thoughts (10). Burnout refers to a reaction to continuous and acute stress at work, including emotional exhaustion, depersonalization, and a decline in personal accomplishment. It is important to note that burnout is not currently classified as an independent diagnosis in either ICD-10 or ICD-11. Instead, it is included as a subtype of “problems related to employment or unemployment” in both classifications. The main differences between the definitions of burnout in ICD-10 and ICD-11 are that the latter emphasizes the role of chronic workplace stress and includes a third dimension related to reduced professional efficacy. This reflects an increased awareness that burnout is a complex phenomenon that is influenced by various individual, organizational, and societal factors (11–14).

Compassion fatigue has been reported to have an impact on both professional and personal life, making it difficult to provide relationship-based nursing care at work and maintain relationships with friends and family members at home. Inability to recognize signs of patients’ symptoms, escalating violence, and an inability to be supportive are signs of compassion fatigue reported by workers in the emergency medicine departments (15). On the other hand, compassion satisfaction includes a positive feeling derived from helping others, finding a purpose in one’s work, and having quality co-worker relationships (16).

Previous empirical evidence has demonstrated the coexistence of both compassion fatigue and compassion satisfaction among caregivers (17–19). It has been found that a high level of exposure to pain and suffering, indicated by an increased number of working days per week, is negatively associated with compassion satisfaction. However, it is positively associated with burnout and secondary traumatic stress (20). Furthermore, a high level of compassion satisfaction has been found to be positively related to a low level of compassion fatigue symptomatology (18, 19). Emergency healthcare workers often experience a wide range of psychological symptoms, including high levels of stress, posttraumatic stress symptoms, burnout, and secondary trauma (21). Previous traumatic experiences, overtime work, and severe occupational stressors, such as resuscitation and death, have been found to be associated with burnout and secondary traumatic stress (22, 23).

Within the category of protective factors, supportive social interactions, physical activity, and the use of meditation have been found to be positively associated with compassion satisfaction (24–28).

Cognitive models of psychopathology suggest that exposure to stressful events can lead to distorted cognitions related to threats, which in turn generate negative emotions and psychopathological reactions such as traumatic stress and depression (29).

In the context of the present study, the stressful events of the pandemic and war were examined to identify their associations with cognitive-emotional processes such as perceived threats and worries, as well as dimensions of professional quality of life including secondary traumatic stress, burnout, and compassion satisfaction.

The pandemic period has been associated with a negative correlation between compassion satisfaction and compassion fatigue among medical staff, as evidenced by Timofeiov-Tudose and Măirean’s (19) research. Additionally, working in a pandemic hospital has been identified as a primary risk factor for secondary trauma among medical staff during the pandemic. Healthcare workers who were closely involved with COVID-19 patients reported higher levels of stress, burnout, and secondary trauma compared to their counterparts who worked with non-COVID-19 patients. However, they also reported higher levels of compassion satisfaction (30–35).

The COVID-19 pandemic has brought about several threats that are specific to this period, including spending time with COVID-19 patients, exposure to patients’ deaths, and experiencing severe COVID-19 infection symptoms in family or friends. These threats have been found to be positively associated with secondary traumatic stress (36). The pandemic has generated concerns about personal and family health, particularly among healthcare workers who are at a higher risk of contracting and spreading the disease to their family members compared to non-healthcare workers (37, 38).

Emergency medicine workers have been particularly affected by the pandemic, experiencing higher levels of burnout compared to other medical specialties (39–41). A previous study revealed that one in two emergency workers had contemplated suicide, and almost half of all emergency medicine workers were deemed to be at high risk for compassion fatigue (42). Medical staff in Romania have also reported high levels of burnout and anxiety during the pandemic (43–46). Factors such as administrative burden, workload, caring for COVID-19 patients, and interpersonal relationships have been linked to an exacerbation of emotional exhaustion among emergency medical staff during the pandemic (47, 48).

Since the outbreak of the Russian-Ukrainian armed conflict, European nations have joined together to provide financial and material support to the Ukrainian government and NGOs operating in the region. Various humanitarian services have also been made available to Ukrainian refugees who have crossed the borders, including free basic health examinations, medical and dental services, counselling services, and free medical treatment for injured military personnel. Medical teams have also been dispatched to border checkpoints to examine travelers and their pets (49, 50).

Empirical evidence has shown that the war has had inevitable implications on the mental health of the Ukrainian people, with high levels of depression, loneliness, fear, nervousness, and anger reported among civilians (51, 52). The threats posed by the conflict in Ukraine have spread to other regions of the world, generating fear, and having a negative impact on the quality of life, particularly in countries closer to military conflicts with the potential to worsen (53, 54). Although exposure to the war was secondary, perceived subjective threat may be a similar or even stronger predictor of stress reactions compared to objective life events (37). Previous empirical evidence has shown that physical proximity to a disaster (e.g., the 2011 Oslo bombing) was related to more posttraumatic stress symptoms (e.g., 55–58). Additionally, exposure to digital content about the Russia-Ukraine war can contribute to amplifying compassion fatigue, regardless of physical proximity to the war zone (59).

The perception of threat can lead to the generation of worry (60). Worries are unpleasant thoughts about future events that imply risk or uncertainty (61) and can generate high levels of anxiety and stress (62). According to the cognitive avoidance theory (60), worries are focused on a possible unwanted event that may happen in the future but is non-existent in the present. Thus, worries are different from threats, which are generated by present events or dangerous situations. Previous research has shown that emergency medical personnel who were exposed to the Russian-Ukrainian armed conflict reported experiencing high levels of anxiety symptoms following the start of the war (45). One source of anxiety may be the worries generated by the risk of the war expanding or the possibility of the Russian Federation using nuclear weapons against Ukraine or another NATO member state. However, to our knowledge, no previous study has assessed how the threat generated by secondary exposure to the ongoing war and worries about being directly affected by the war are associated with professional quality of life.

### 1.1. The present study

Given previous research findings that have highlighted the threats posed by the COVID-19 pandemic and armed conflict (e.g., 31, 45, 63), the objective of the present study is to identify the associations between these concurrent threats during a period marked by the outbreak of war and the ongoing threat of the pandemic, and indicators of professional quality of life, namely secondary traumatic stress, burnout, and compassion satisfaction. We aim to identify these relationships due to the limited literature on war-related factors in relation to compassion fatigue, and the absence of previous research on how vicarious trauma resulting from war may be associated with compassion satisfaction. While previous studies have explored the link between secondary exposure to trauma and quality of life, none have provided empirical evidence of the relationship between secondary exposure to the Russian-Ukrainian armed conflict and quality of life. Identifying both the threats generated by secondary exposure to war trauma and concerns about potential direct exposure to war conditions or consequences will help us understand factors related to quality of life. Additionally, we aim to determine whether the pandemic continues to pose a threat at the time of the study and whether it can still predict indicators of professional quality of life. Based on previous results (e.g., 31, 42, 45), we anticipate that both the threats generated by the war and the pandemic, as well as daily worries, will positively predict secondary traumatic stress and burnout, and will negatively predict compassion satisfaction.

## 2. Method

### 2.1. Participants

The study included a sample of 372 emergency medical personnel staff from the emergency units of five county hospitals, including a university center, located in the Eastern region of Romania, bordering Ukraine, and Moldova. The inclusion criteria for the study required participants to be medical personnel working in the emergency department of a hospital. Of the total sample, 56.7% were nurses and 43.3% were physicians. Most of the sample was comprised of women (77.2%). Participants’ ages ranged from 22 to 66 years old (*M* age = 39.41; SD = 9.84), and their years of professional experience ranged from less than a year to 35 years, with an average of 10.48 (SD = 8.82). The healthcare workforce in Romania is predominantly female, with women making up 77.7% of the workforce according to Eurostat data from 2021. In specific medical professions, such as midwifery, nursing, and physician roles, women make up 96.2%, 91.1%, and 67.4% respectively, which is higher than the EU averages for these professions. Therefore, the predominance of female respondents in our study aligns with the gender distribution of medical workers in Romania (64, 65).

### 2.2. Measurements

The measurement of **perceived threats** was conducted using four items that assessed threats in the domains of health, economics, security, and politics (66). Each item was evaluated twice, once in relation to the Russian-Ukrainian war and once in relation to the COVID-19 pandemic. The items were rated on a 5-point Likert scale, ranging from 1 (“not threatening at all”) to 5 (“threatening very much”). Two total scores were calculated for war threats (*α* = 0.82) and pandemic threats (*α* = 0.84) by summing the responses. Higher scores indicated higher levels of perceived threat.

The study aimed to measure **the daily worries arising from war** using a five-item questionnaire that assessed concerns related to financial instability, personal safety, family safety, and job security in the context of the war outbreak. Participants rated each item on a 5-point Likert scale ranging from 1 (not at all worried) to 5 (extremely worried). A composite score was computed by summing the responses, with a high internal consistency (*α* = 0.92), indicating that the items reliably measured a single construct. Higher scores on the composite score indicated greater levels of war-related worries.

**The Professional Quality of Life Scale**, ProQOL (7), Romanian version (18, 19), was used to measure the professional quality of life. The scale consists of 30 items that assess three domains: compassion satisfaction (*α* = 0.80), burnout (*α* = 0.67), and secondary traumatic stress (*α* = 0.78). Participants rated each item on a 5-point Likert scale ranging from 1 (not at all) to 5 (always). Total scores were calculated by summing the responses, with higher scores indicating higher levels of secondary traumatic stress, burnout, and compassion satisfaction.

In addition, a **demographic questionnaire** was administered to collect information on age, gender, number of years of professional experience, and professional category (e.g., nurses and physicians).

### 2.3. Procedure

The study invitation was distributed via email to all medical workers in the emergency departments of northeastern cities in Romania. In the first step, the invitation was sent to the section heads, who then contacted their team members and invited them to participate in the study on a voluntary basis. The main objective of the study was to assess the perceptions of daily workplace challenges and quality of life among emergency department workers. Participants were informed that their participation was voluntary and that their answers would be kept confidential. After providing informed consent, participants completed an online survey that took approximately 15 min. Data were collected in early March 2022, shortly after the outbreak of the Russian-Ukrainian armed conflict. Participation was not remunerated.

## 3. Results

### 3.1. Preliminary analysis

The independent samples t-test revealed non-significant gender differences in secondary traumatic stress (STS), burnout, and compassion satisfaction, all *p* > 0.05. However, there were significant differences between professional categories (i.e., nurses, physicians) in STS, *t*(360) = 4.89, *p* < 0.001, burnout, *t*(364) = 3.95, *p* < 0.001, and compassion satisfaction, *t*(358) = −3.67, *p* < 0.001. Nurses reported lower levels of STS (*M* = 22.50, SD = 6.63) and burnout (*M* = 22.40, SD = 5.06), and higher levels of compassion satisfaction (*M* = 43.66, SD = 5.58), compared to physicians (*M* = 25.95, SD = 6.70; *M* = 24.59, SD = 5.50; *M* = 41.26, SD = 6.75, respectively). These results are presented in Table 1.

**Table 1:** Differences between physicians and nurses concerning the study variables.

Variables	Professional category				*t*	*d*
	Physicians (*N* = 161)		Nurses (*N* = 211)			
	Mean	SD	Mean	SD		
1. Threat pandemic	8.75	3.08	7.60	3.42	3.35**	0.35
2. Threat war	12.72	2.49	11.26	3.32	4.84***	0.50
3. Daily worries	19.11	4.62	16.98	5.40	4.01***	0.41
4. Secondary trauma	25.95	6.70	22.50	6.63	4.89***	0.51
5. Burnout	24.59	5.50	22.40	5.06	3.95***	0.41
6. Compassion satisfaction	41.26	6.72	43.66	5.58	−3.67***	0.38
7. Age	38.08	9.65	40.42	9.90	−2.27*	0.23
8. Professional experience	8.35	7.58	12.22	9.61	−4.16***	0.43

*p < 0.05; **p < 0.01; ***p < 0.001. *N* = 372.

Age was negatively related to perceived threat related to war, STS, and burnout, and positively related to compassion satisfaction.

### 3.2. Associations among the main study variables

Pearson correlations revealed that both secondary traumatic stress (STS) and burnout were positively associated with perceived threats related to the pandemic and war, as well as daily worries. In contrast, compassion satisfaction was negatively associated with perceived threats related to war and daily worries. Compassion satisfaction was not significantly related to perceived threats related to the pandemic. These findings are presented in Table 2.

**Table 2:** Zero-order correlations between the main study variables.

Variables	1	2	3	4	5	6	7
1. Threat pandemic							
2. Threat war	0.56***						
3. Daily worries	0.53***	0.82***					
4. Secondary trauma	0.35***	0.37***	0.43***				
5. Burnout	0.27***	0.35***	0.37***	0.71***			
6. Compassion satisfaction	−0.08	−0.19***	−0.18***	−0.33***	−0.70***		
7. Experience	−0.04	−0.09	−0.06	−0.09	−0.09	0.04	
8. Age	−0.04	−0.10*	−0.08	−0.10*	−0.18***	−0.15**	0.70***
Mean	8.10	11.89	17.90	24.02	23.34	42.62	10.48
SD	3.32	3.07	5.18	6.87	5.36	6.22	8.82
Range	3–15	4–15	5–24	12–50	12–38	20–50	0–35

*p < 0.05; **p < 0.01; ***p < 0.001. *N* = 372.

### 3.3. Regression analyses for the predictors of compassion fatigue and compassion satisfaction

To examine the extent to which perceived threats and daily worries explained variance in participants’ secondary traumatic stress (STS), burnout, and compassion satisfaction, three hierarchical multiple regression analyses were conducted. In each analysis, STS (regression 1), burnout (regression 2), and compassion satisfaction (regression 3) were the dependent variables. Demographic variables, including age and profession, were entered in Step 1. Perceived threats related to the pandemic and war, as well as daily worries, were entered in Step 2.

Results indicated that STS was positively predicted by perceived threats related to the pandemic and daily worries. The final model explained 21.5% of the variance in STS. Burnout was positively predicted only by daily worries, and the model with all predictors explained 16.5% of the variance. Finally, compassion satisfaction was not predicted by the variables entered in the analysis. The results are presented in Table 3.

**Table 3:** Hierarchical linear regression analysis for compassion fatigue and compassion satisfaction.

	Compassion fatigue						Compassion satisfaction		
	Secondary traumatic stress			Burnout					
	*B*	SE	*β*	*B*	SE	*β*	*B*	SE	*β*
*Step 1: Demographics*									
Age	−0.09	0.03	−0.13*	−0.11	0.02	−0.20***	0.10	0.03	0.16**
Profession	1.04	0.32	0.16**	0.62	0.25	0.12*	−0.64	0.29	−0.11*
*R* ^2^	0.034**			0.045***			0.031**		
Δ*R*^2^	0.040***			0.050***			0.036***		
*Step 2*									
Age	−0.06	0.03	−0.09*	−0.08	0.02	−0.16**	0.09	0.03	0.14**
Profession	0.064	0.29	0.10*	0.31	0.24	0.06	−0.47	0.29	−0.08
Threats pandemic	0.36	0.12	0.17**	0.14	0.09	0.08	0.07	0.11	0.04
Threats war	−0.11	0.19	−0.05	0.16	0.15	0.09	−0.21	0.19	−0.10
Daily worries	0.48	0.11	0.36***	0.22	0.09	0.22*	−0.11	0.11	−0.09
*R* ^2^	0.215***			0.165***			0.052***		
Δ*R*^2^	0.186***			0.126***			0.030*		

*p < 0.05. **p < 0.01. ***p < 0.001.

## 4. Discussion

The aim of the present study was to investigate the relationship between threats generated by the pandemic and the outbreak of the Russian-Ukrainian war, as well as daily worries, with dimensions of professional quality of life, including secondary traumatic stress, burnout, and compassion satisfaction. The study sample consisted of emergency medicine personnel, and data were collected soon after the outbreak of the Russian-Ukrainian conflict.

Contrary to our expectations based on previous literature (37, 54, 58), our results indicated that war threats did not predict any dimension of professional quality of life. However, in accordance with previous empirical findings (39, 40, 42), perceived threats related to the pandemic positively predicted secondary traumatic stress. Therefore, consistent with the definition of compassion fatigue (8, 9), our results suggest that cumulative exposure during a prolonged period, such as the pandemic period, may increase the risk of secondary traumatic stress. Although many of the restrictions imposed by the pandemic were no longer in place when the present study was conducted, threats generated by the pandemic still showed associations with unwanted psychological outcomes, such as secondary traumatic stress. Threats generated by the war did not demonstrate a direct relationship with any of the indicators of professional quality of life, but daily worries generated by the war positively predicted both secondary traumatic stress and burnout. Some previous evidence [e.g., (45)] has shown that high anxiety experienced after the outbreak of war was related to poor general health. These results may be explained by the cognitive avoidance theory of worry (60), which suggests that threats predict worries. Thus, anticipated risk of being directly affected by the war, rather than perceived threats of secondary exposure in the present, is a more proximal predictor of professional quality of life. Future longitudinal studies could further explore the mediating role of worries about being directly affected by a trauma in the relation between secondary trauma and future outcomes, including positive and negative indicators of quality of life.

Regarding these results, two important aspects should be noted. First, both the pandemic, which involved cumulative exposure, and the war, which involved a low and more distant level of exposure, had the potential to generate worries and predict low quality of life. These results highlight the increased risks for this professional category and the need to raise awareness about the risks in order to prevent a decrease in professional quality of life. Second, our results did not identify any relationship between threats, worries, and compassion satisfaction. Thus, this positive indicator of quality of life remained constant despite threats and worries.

Given the strong negative associations between compassion satisfaction and secondary traumatic stress and burnout, as shown by our results, compassion satisfaction can be considered a valuable resource for attenuating the negative implications of professional challenges. Improving the ability to recognize satisfaction from using personal abilities and expertise in saving lives could prevent traumatic stress and burnout in the long term.

From a practical standpoint, the results could contribute to raising awareness about the potential implications of different consecutive and concurrent challenges, such as the war and the pandemic. As many studies have documented, compassion fatigue affects both professional and personal quality of life, and medical field employers are vulnerable to high levels of burnout and secondary trauma, the two components of compassion fatigue. A first step in preventing the development of high levels of symptomatology is to be aware of the phenomenon and its implications. Thus, psychoeducation about self-recognition of early signs of compassion fatigue is necessary and could be both a personal and organizational responsibility. Along with awareness, professional boundaries, self-care practices (e.g., hobbies, healthy sleep patterns, healthy eating, physical activity, breathing exercises, etc.), and education on the subject at the individual and organizational levels could help prevent compassion fatigue (67–69). Supervision may be particularly beneficial for employers with low levels of professional experience to prevent exhaustion and secondary trauma (8). Formal debriefing and social support could also be useful coping mechanisms against secondary traumatic stress (70). Interventions such as Mindful Self-Compassion (MSC) training enhance coping strategies like mindfulness and compassion satisfaction, and they also reduce secondary traumatic stress and burnout in trauma personnel (71).

Several limitations should be noted. First, the cross-sectional design of the study does not allow us to draw conclusions in terms of causal relations between variables. Thus, we cannot be sure that threats and worries lead to low professional quality of life. However, the results collected soon after the war outbreak offer us a picture of the potential impact on people’s lives, who were secondary exposed to others’ traumas. Second, the sample is highly comprised of women, and the possibility to generalize the results is limited. Third, the present study did not identify specific worries generated by the war. Analyzing which type of worries (e.g., related to personal health, family health, safety, and financial difficulties) better explains STS and burnout would be more informative from a practical standpoint. Both scientists and practitioners could benefit from knowing what specific worries create vulnerability to secondary traumatic stress and burnout.

Despite these limitations, the present study has important implications for understanding professional quality of life during very challenging life events. One of the most important results is that, although the risks are lower, an event with direct involvement (i.e., pandemic) is associated with detrimental outcomes compared with a riskier event but with indirect exposure. Furthermore, this is one of the few studies that documented the implications of the Russian-Ukrainian war on mental health and, as far as we know, the first one that concurrently assessed two important sources of burnout and stress (i.e., pandemic and war) among medical staff. Another strength of the present study is that it examined both positive (i.e., compassion satisfaction) and negative (i.e., compassion fatigue) dimensions of professional quality of life, contributing to a deeper understanding of the phenomenon.

In conclusion, the present study highlighted the associations of pandemic threats and daily worries generated by the war with professional quality of life in a sample of emergency medicine practitioners. The results found positive associations between threats, worries, and negative dimensions of quality of life (i.e., secondary traumatic stress, and burnout). The relations of threats and worries with compassion satisfaction were non-significant. Future studies could explore personal and organizational factors that increase vulnerability to secondary traumatic stress and burnout.

## Data availability statement

The datasets presented in this study can be found in online repositories. The names of the repository/repositories and accession number(s) can be found at: https://doi.org/10.5281/zenodo.7840114.

## Ethics statement

The studies involving human participants were reviewed and approved by Ethics Committee of Saint Spiridon County Hospital Iasi (approval code: 23, approval date: March 11, 2022). The patients/participants provided their written informed consent to participate in this study.

## Author contributions

AH, CM, and DC contributed to the conception and design of the study and wrote the first draft. SH wrote the first draft of the manuscript. M-OP organized the database and wrote the first draft. CM performed the statistical analysis. SV, MH, and CK wrote sections of the manuscript. All authors contributed to the article and approved the submitted version.

## Conflict of interest

The authors declare that the research was conducted in the absence of any commercial or financial relationships that could be construed as a potential conflict of interest.

## Publisher’s note

All claims expressed in this article are solely those of the authors and do not necessarily represent those of their affiliated organizations, or those of the publisher, the editors and the reviewers. Any product that may be evaluated in this article, or claim that may be made by its manufacturer, is not guaranteed or endorsed by the publisher.

## References

[1] Cîrstoveanu C Oprea B Burtăverde V Dimitriu M Stoian PA Ionescu AC. Emotional intelligence and the perception of stressors at work among healthcare employees in neonatology and paediatrics. Mediterr J Clin Psychol. (2020) 8:1–14. doi: 10.6092/2282-1619/mjcp-2678

[2] Petrino R Riesgo LG Yilmaz B. Burnout in emergency medicine professionals after 2 years of the COVID-19 pandemic: a threat to the healthcare system? Eur J Emerg Med. (2022) 29:279–84. doi: 10.1097/MEJ.0000000000000952, PMID: 35620812 PMC9241557

[3] Adelman RD Tmanova LL Delgado D Dion S Lachs MS. Caregiver burden: a clinical review. JAMA. (2014) 311:1052–60. doi: 10.1001/jama.2014.304 24618967

[4] Bevans M Sternberg EM. Caregiving burden, stress, and health effects among family caregivers of adult cancer patients. JAMA. (2012) 25:398–403. doi: 10.1001/jama.2012.29, PMID: 22274687 PMC3304539

[5] Merlo EM Stoian AP Motofei IG Settineri S. Clinical psychological figures in healthcare professionals: resilience and maladjustment as the “cost of care”. Front Psychol. (2020) 1:607783. doi: 10.3389/fpsyg.2020.607783, PMID: 33335503 PMC7736062

[6] Moeller SD. Compassion fatigue: How the media sell disease, famine, war and death. 1st ed. New York: Routledge (1999).

[7] Stamm BH. The concise pro QOL manual. Pocatello: (2010) ID: The ProQOL.org.

[8] Boublil E. Compassion fatigue: assessing the psychological and moral boundaries of empathy In: Phenomenology of bioethics: technoethics and lived-experience Springer (2021). 61–72. Available at: https://philpapers.org/rec/FERPOB-4.

[9] Figley CR. Compassion fatigue as secondary traumatic stress disorder: an overview In: Figley CR, editor. Compassion fatigue: Coping with secondary traumatic stress disorder. New York, New York: Brunner/Mazel (1995)

[10] Figley CR In: Stamm BH, editor. Secondary traumatic stress: self-care issues for clinicians, researchers: The Sidran Press (1995)

[11] ICD-10 Version: (2019). Available at: https://icd.who.int/browse10/2019/en#/Z73.0 (Accessed June 14, 2023)

[12] World Health Organization ICD-11. Available at: https://www.who.int/standards/classifications/classification-of-diseases (Accessed June 14, 2023)

[13] AMA. WHO adds burnout to ICD-11. What it means for physicians. Available at: https://www.ama-assn.org/practice-management/physician-health/who-adds-burnout-icd-11-what-it-means-physicians (Accessed June 14, 2023)

[14] Maslach C Jackson SE. The measurement of experienced burnout. J Organ Behav. (1981) 2:99–113. doi: 10.1002/job.4030020205, PMID: 37428737

[15] Wolf LA Delao AM Perhats C Clark PR Edwards C Frankenberger WD. Traumatic stress in emergency nurses: does your work environment feel like a war zone? Int Emerg Nurs. (2020) 52:100895. doi: 10.1016/j.ienj.2020.100895, PMID: 32795958

[16] Sodeke-Gregson EA Holttum S Billings J. Compassion satisfaction, burnout, and secondary traumatic stress in UK therapists who work with adult trauma clients. Eur J Psychotraumatol. (2013) 4:21869. doi: 10.3402/ejpt.v4i0.21869, PMID: 24386550 PMC3877781

[17] Owen RP Wanzer L. Compassion fatigue in military healthcare teams. Arch Psychiatr Nurs. (2014) 28:2–9. doi: 10.1016/j.apnu.2013.09.007, PMID: 24506980

[18] Măirean C. Emotion regulation strategies, secondary traumatic stress, and compassion satisfaction in healthcare providers. J Psychol. (2016) 16:961–75. doi: 10.1080/00223980.2016.1225659, PMID: 27629057

[19] Timofeiov-Tudose IG Măirean C. Workplace humour, compassion, and professional quality of life among medical staff. Eur J Psychotraumatol. (2023) 14:2158533. doi: 10.1080/20008066.2022.2158533, PMID: 37052083 PMC9793908

[20] Merlo EM McNabney SM Frisone F Sicari F Paunica M Motofei C. Compassion and suppression in caregivers: twin masks of tragedy and joy of caring. J Mind Med Sci. (2020) 7:1. doi: 10.22543/7674.71.P6168

[21] Alanazi TNM McKenna L Buck M Alharbi RJ. Reported effects of the COVID-19 pandemic on the psychological status of emergency healthcare workers: a scoping review. Australas Emerg Care. (2022) 25:197–212. doi: 10.1016/j.auec.2021.10.002, PMID: 34802977 PMC8585598

[22] Hinderer KA VonRueden KT Friedmann E McQuillan KA Gilmore R Kramer B. Burnout, compassion fatigue, compassion satisfaction, and secondary traumatic stress in trauma nurses. J Trauma Nurs. (2014) 21:160–9. doi: 10.1097/JTN.0000000000000055, PMID: 25023839

[23] Roden-Foreman JW Bennett MM Rainey EE Garrett JS Powers MB Warren AM. Secondary traumatic stress in emergency medicine clinicians. Cogn Behav Ther. (2017) 46:522–32. doi: 10.1080/16506073.2017.1315612, PMID: 28452256

[24] Hunsaker S Chen H-C Maughan D Heaston S. Factors that influence the development of compassion fatigue, burnout, and compassion satisfaction in emergency department nurses: compassion fatigue, satisfaction, and burnout. J Nurs Scholarsh. (2015) 47:186–94. doi: 10.1111/jnu.12122, PMID: 25644276

[25] Zhang Y-Y Zhang C Han X-R Li W Wang Y-L. Determinants of compassion satisfaction, compassion fatigue and burn out in nursing: a correlative meta-analysis. Medicine (Baltimore). (2018) 97:e11086. doi: 10.1097/MD.0000000000011086, PMID: 29952947 PMC6242309

[26] Hundall Stamm B. (2009). Professional quality of life measure: Compassion, satisfaction, and fatigue version 5. Pro QOL. Available at: https://proqol.org/ProQol_Test.html

[27] Makic MBF. Taking Care of the Caregiver: compassion satisfaction and compassion fatigue. J Perianesth Nurs. (2015) 30:546–7. doi: 10.1016/j.jopan.2015.09.006, PMID: 26596389

[28] Velutti L Pavesi C Poggio C Carettoni B Saetta A Arcanà C. Caregiver stress: clinical evaluation and intervention strategies for caregiver burden prevention. J Clin Oncol. (2017) 35:22. doi: 10.1200/JCO.2017.35.31_suppl.22

[29] Dobson KS Poole JC Beck JS. The fundamental cognitive model In: Leahy RL, editor. Science and practice in cognitive therapy: foundations, mechanisms, and applications. New York: Guilford Press (2018). 29–47.

[30] Giusti EM Pedroli E D’Aniello GE Stramba Badiale C Pietrabissa G Manna C. The psychological impact of the COVID-19 outbreak on health professionals: a cross-sectional study. Front Psychol. (2020) 10:1684. doi: 10.3389/fpsyg.2020.01684, PMID: 32754102 PMC7366071

[31] Trumello C Bramanti SM Ballarotto G Candelori C Cerniglia L Cimino S. Psychological adjustment of healthcare workers in Italy during the COVID-19 pandemic: differences in stress, anxiety, depression, burnout, secondary trauma, and compassion satisfaction between frontline and non-frontline professionals. Int J Environ Res Public Health. (2020) 17:8358. doi: 10.3390/ijerph17228358, PMID: 33198084 PMC7696387

[32] Almeida M. Burnout and the mental health impact of COVID-19 in anesthesiologists: a call to action. J Clin Anesth. (2021) 68:110084. doi: 10.1016/j.jclinane.2020.110084, PMID: 33038719 PMC7524672

[33] Blekas A Voitsidis P Athanasiadou M Parlapani E Chatzigeorgiou AF Skoupra M. COVID-19: PTSD symptoms in Greek health care professionals. Psychol Trauma. (2020) 12:812–9. doi: 10.1037/tra0000914, PMID: 32853011

[34] Bria M Baban A Dumitrascu D. Systematic review of burnout risk factors among European healthcare professionals. Cogn Brain Behav Interdiscip J. (2012) 16:423–52.

[35] Chen Q Liang M Li Y Guo J Fei D Wang L. Mental health care for medical staff in China during the COVID-19 outbreak. Lancet Psychiatry. (2020) 7:e15–6. doi: 10.1016/S2215-0366(20)30078-X, PMID: 32085839 PMC7129426

[36] Orrù G Marzetti F Conversano C Vagheggini G Miccoli M Ciacchini R. Secondary traumatic stress and burnout in healthcare workers during COVID-19 outbreak. Int J Environ Res Public Health. (2021) 18:337. doi: 10.3390/ijerph18010337, PMID: 33466346 PMC7794988

[37] Ozen G Zanfardino A Ozen G Acan B Piscopo A Casaburo F. Comparison of emotional approaches of medical doctors against COVID-19 pandemic: eastern and Western Mediterranean countries. Int J Clin Pract. (2021) 75:e14973. doi: 10.1111/ijcp.14973, PMID: 34626512 PMC8646585

[38] Di Giuseppe M Nepa G Prout TA Albertini F Marcelli S Orrù G. Stress, Burnout, and resilience among healthcare workers during the COVID-19 emergency: the role of defense mechanisms. Int J Environ Res Public Health. (2021) 14:5258. doi: 10.3390/ijerph18105258, PMID: 34069270 PMC8156145

[39] Lopez J Bindler RJ Lee J. Cross-sectional analysis of burnout, secondary traumatic stress, and compassion satisfaction among emergency nurses in Southern California working through the COVID-19 pandemic. J Emerg Nurs. (2022) 48:366–375.e2. doi: 10.1016/j.jen.2022.03.008, PMID: 35690484 PMC8958096

[40] McKinley N McCain RS Convie L Clarke M Dempster M Campbell WJ. Resilience, burnout and coping mechanisms in UK doctors: a cross-sectional study. BMJ Open. (2020) 10:e031765. doi: 10.1136/bmjopen-2019-031765, PMID: 31988223 PMC7045750

[41] Di Trani M Mariani R Ferri R De Berardinis D Frigo MG. From resilience to burnout in healthcare workers during the COVID-19 emergency: the role of the ability to tolerate uncertainty. Front Psychol. (2021) 16:646435. doi: 10.3389/fpsyg.2021.646435, PMID: 33935905 PMC8085585

[42] Renkiewicz GK Hubble MW. Secondary traumatic stress in emergency services systems (STRESS) project: quantifying and predicting compassion fatigue in emergency medical services personnel. Prehosp Emerg Care. (2022) 26:652–63. doi: 10.1080/10903127.2021.1943578, PMID: 34128453

[43] Corlade-Andrei M Măirean C Nedelea P Grigorași G Cimpoeșu D. Burnout syndrome among staff at an emergency department during the COVID-19 pandemic. Healthcare (Basel). (2022) 10:258. doi: 10.3390/healthcare10020258, PMID: 35206873 PMC8872313

[44] Dimitriu MCT Pantea-Stoian A Smaranda AC Nica AA Carap AC Constantin VD. Burnout syndrome in Romanian medical residents in time of the COVID-19 pandemic. Med Hypotheses. (2020) 144:109972. doi: 10.1016/j.mehy.2020.109972, PMID: 32531540 PMC7276114

[45] Hăisan A Măirean C Lupuşoru SI Tărniceriu C Cimpoeşu D. General health among eastern Romanian emergency medicine personnel during the Russian-Ukrainian armed conflict. Healthcare (Basel). (2022) 10:1976. doi: 10.3390/healthcare10101976, PMID: 36292423 PMC9602120

[46] Sima R-M Olaru O-G Cazaceanu A Scheau C Dimitriu M-T Popescu M. Stress and anxiety among physicians and nurses in Romania during the COVID-19 pandemic. J Mind Med Sci. (2021) 8:252–8. doi: 10.22543/7674.82.P252258, PMID: 35885687

[47] Moscu C-A Anghele M Dragomir L Munteanu S Anghele A Nechita A. Emotional exhaustion and professional satisfaction during covid-19 pandemic at the level of emergency department staff. Brain (Bacau). (2021) 12:265–78. doi: 10.18662/brain/12.2/205

[48] Tarchi L Crescenzo P Talamonti K. Prevalence and predictors of mental distress among Italian red cross auxiliary corps: a cross-sectional evaluation after deployment in anti-COVID-19 operations. Mil Psychol. (2022):1–14. doi: 10.1080/08995605.2022.2069983 37615558 PMC10453978

[49] World Health Organization, Ukraine Crisis. (1970). Public Health Situation Analysis: Refugee-Hosting Countries, 17 March 2022. World Health Organization. Retrieved, January 1. from, Available at: https://apps.who.int/iris/handle/10665/352494. (Accessed April 29, 2022).

[50] Jain N Prasad S Bordeniuc A Tanasov A Shirinskaya AV Béla B. European countries step-up humanitarian and medical assistance to Ukraine as the conflict continues. J Prim Care Community Health. (2022) 13:21501319221095360. doi: 10.1177/21501319221095358, PMID: 35465746 PMC9036314

[51] Chaaya C Devi Thambi V Sabuncu Ö Abedi R Osman Ahmed Osman A Uwishema O. Ukraine-Russia crisis and its impacts on the mental health of Ukrainian young people during the COVID-19 pandemic. Ann Med Surg (Lond). (2022) 79:104033. doi: 10.1016/j.amsu.2022.104033, PMID: 35765517 PMC9221679

[52] Kurapov A Pavlenko V Drozdov A Bezliudna V Reznik A Isralowitz R. Toward an understanding of the Russian-Ukrainian war impact on university students and personnel. J Loss Trauma. (2022) 1–8. doi: 10.1080/15325024.2022.2084838

[53] Tyson J. Compassion fatigue in the treatment of combat-related trauma during wartime. Clin Soc Work J. (2007) 35:183–92. doi: 10.1007/s10615-007-0095-3

[54] Mărcău FC Peptan C Gorun HT Băleanu VD Gheorman V. Analysis of the impact of the armed conflict in Ukraine on the population of Romania. Front Public Health. (2022) 10:964576. doi: 10.3389/fpubh.2022.964576, PMID: 35968467 PMC9372605

[55] Abu Suhaiban H Grasser LR Javanbakht A. Mental health of refugees and torture survivors: a critical review of prevalence, predictors, and integrated care. Int J Environ Res Public Health. (2019) 28:2309. doi: 10.3390/ijerph16132309, PMID: 31261840 PMC6651013

[56] Hynes H. On the battlefield of women’s bodies: an overview of the harm of war to women. Women’s Stud Int Forum. (2004) 27:431–45. doi: 10.1016/j.wsif.2004.09.001

[57] Yehuda R Lehrner A. Intergenerational transmission of trauma effects: putative role of epigenetic mechanisms. World Psychiatry. (2018) 17:243–57. doi: 10.1002/wps.20568, PMID: 30192087 PMC6127768

[58] Heir T Blix I Knatten CK. Thinking that one’s life was in danger: perceived life threat in individuals directly or indirectly exposed to terror. Br J Psychiatry. (2016) 209:306–10. doi: 10.1192/bjp.bp.115.170167, PMID: 27056624

[59] Zhan J. (2022). The relevance of compassion fatigue in social media discourse on the Russia-Ukraine crisis. In 2022 5th international conference on humanities education and social sciences (ICHESS 2022) Atlantis Press. pp. 298–311. Available at: https://www.atlantis-press.com/proceedings/ichess-22/125983072.

[60] Sibrava NJ Borkovec TD. The cognitive avoidance theory of worry In: Davey G. C. L. Wells A. (Eds.), Worry and its psychological disorders: Theory, assessment and treatment. Willey (2006). 239–56. doi: 10.1002/9780470713143.ch14

[61] Mantzios M. Exploring the relationship between worry and impulsivity in military recruits: the role of mindfulness and self-compassion as potential mediators: worry, impulsivity, mindfulness, self-compassion. Stress Health. (2014) 30:397–404. doi: 10.1002/smi.2617, PMID: 25476964

[62] Zysberg L Zisberg A. Days of worry: emotional intelligence and social support mediate worry in the COVID-19 pandemic. J Health Psychol. (2022) 27:268–77. doi: 10.1177/1359105320949935, PMID: 32811195

[63] Head M., (2022). Ukraine: Disease control is a casualty of war – So a surge in COVID cases is likely, the conversation. Retrieved April 17, 2022, Available at: https://theconversation.com/amp/kraine-disease-control-is-a-casualty-of-war-so-a-surge-in-covid-cases-is-likely-179218. (Accessed June 14, 2023)

[64] Eurostat. (2021). Physicians by sex, age and specialty. Available at: https://appsso.eurostat.ec.europa.eu/nui/show.do?dataset=hlth_rs_spec&lang=en (Accessed June 14, 2023)

[65] National Institute of Statistics. (2021). Women and men in Romania. Available at: https://insse.ro/cms/ro/content/femei-si-barbati-in-romania-0 (Accessed June 14, 2023)

[66] Marciano H Eshel Y Kimhi S Adini B. Hope and fear of threats as predictors of coping with two major adversities, the COVID-19 pandemic and an armed conflict. Int J Environ Res Public Health. (2022) 19:1123. doi: 10.3390/ijerph19031123, PMID: 35162144 PMC8834741

[67] İlhan B Küpeli İ. Secondary traumatic stress, anxiety, and depression among emergency healthcare workers in the middle of the COVID-19 outbreak: a cross-sectional study. Am J Emerg Med. (2022) 52:99–104. doi: 10.1016/j.ajem.2021.11.051, PMID: 34894474 PMC8641431

[68] Peters E. Compassion fatigue in nursing: a concept analysis. Nurs Forum. (2018) 53:466–80. doi: 10.1111/nuf.12274, PMID: 29962010

[69] Xu HG Kynoch K Tuckett A Eley R. Effectiveness of interventions to reduce emergency department staff occupational stress and/or burnout: a systematic review: a systematic review. JBI Evid Synth. (2020) 18:1156–88. doi: 10.11124/JBISRIR-D-19-00252, PMID: 32813371

[70] Morrison LE Joy JP. Secondary traumatic stress in the emergency department. J Adv Nurs. (2016) 72:2894–906. doi: 10.1111/jan.13030, PMID: 27221701

[71] Delaney MC. Caring for the caregivers: evaluation of the effect of an eight-week pilot mindful self-compassion (MSC) training program on nurses’ compassion fatigue and resilience. PLoS One. (2018) 13:e0207261. doi: 10.1371/journal.pone.0207261, PMID: 30462717 PMC6248952

